# Damage to Broca’s area does not contribute to long-term speech production outcome after stroke

**DOI:** 10.1093/brain/awaa460

**Published:** 2021-01-31

**Authors:** Andrea Gajardo-Vidal, Diego L Lorca-Puls, PLORAS team, Holly Warner, Bawan Pshdary, Jennifer T Crinion, Alexander P Leff, Thomas M H Hope, Sharon Geva, Mohamed L Seghier, David W Green, Howard Bowman, Cathy J Price

**Affiliations:** 1 Wellcome Centre for Human Neuroimaging, UCL Queen Square Institute of Neurology, London, UK; 2 Faculty of Health Sciences, Universidad del Desarrollo, Concepcion, Chile; 3 Department of Speech, Language and Hearing Sciences, Faculty of Medicine, Universidad de Concepcion, Concepcion, Chile; 4 Institute of Cognitive Neuroscience, University College London, London, UK; 5 Department of Brain Repair and Rehabilitation, UCL Queen Square Institute of Neurology, London, UK; 6 Cognitive Neuroimaging Unit, Emirates College for Advanced Education, Abu Dhabi, UAE; 7 Department of Biomedical Engineering, Khalifa University of Science and Technology, Abu Dhabi, UAE; 8 Department of Experimental Psychology, University College London, London, UK; 9 Centre for Cognitive Neuroscience and Cognitive Systems and the School of Computing, University of Kent, Canterbury, UK; 10 School of Psychology, University of Birmingham, Birmingham, UK

**Keywords:** Broca’s area, arcuate fasciculus, speech production, aphasia, stroke

## Abstract

Broca’s area in the posterior half of the left inferior frontal gyrus has long been thought to be critical for speech production. The current view is that long-term speech production outcome in patients with Broca’s area damage is best explained by the combination of damage to Broca’s area and neighbouring regions including the underlying white matter, which was also damaged in Paul Broca’s two historic cases. Here, we dissociate the effect of damage to Broca’s area from the effect of damage to surrounding areas by studying long-term speech production outcome in 134 stroke survivors with relatively circumscribed left frontal lobe lesions that spared posterior speech production areas in lateral inferior parietal and superior temporal association cortices. Collectively, these patients had varying degrees of damage to one or more of nine atlas-based grey or white matter regions: Brodmann areas 44 and 45 (together known as Broca’s area), ventral premotor cortex, primary motor cortex, insula, putamen, the anterior segment of the arcuate fasciculus, uncinate fasciculus and frontal aslant tract. Spoken picture description scores from the Comprehensive Aphasia Test were used as the outcome measure. Multiple regression analyses allowed us to tease apart the contribution of other variables influencing speech production abilities such as total lesion volume and time post-stroke. We found that, in our sample of patients with left frontal damage, long-term speech production impairments (lasting beyond 3 months post-stroke) were solely predicted by the degree of damage to white matter, directly above the insula, in the vicinity of the anterior part of the arcuate fasciculus, with no contribution from the degree of damage to Broca’s area (as confirmed with Bayesian statistics). The effect of white matter damage cannot be explained by a disconnection of Broca’s area, because speech production scores were worse after damage to the anterior arcuate fasciculus with relative sparing of Broca’s area than after damage to Broca’s area with relative sparing of the anterior arcuate fasciculus. Our findings provide evidence for three novel conclusions: (i) Broca’s area damage does not contribute to long-term speech production outcome after left frontal lobe strokes; (ii) persistent speech production impairments after damage to the anterior arcuate fasciculus cannot be explained by a disconnection of Broca’s area; and (iii) the prior association between persistent speech production impairments and Broca’s area damage can be explained by co-occurring white matter damage, above the insula, in the vicinity of the anterior part of the arcuate fasciculus.

## Teaser

Contrary to the dominant view that speech production impairments after a left frontal lobe stroke involving Broca’s area are caused by the combination of damage to Broca’s area and neighbouring regions, Gajardo-Vidal *et al*. show that Broca’s area damage does not in fact contribute to long-term speech production outcomes.

## Introduction

For over 150 years, clinical aphasiology and behavioural neurology have been fundamentally influenced by Paul Broca’s finding that stroke survivors with severe and persistent speech production impairments had damage to the third convolution of the left frontal lobe (Broca, 1861*a*, *b*, 1865). Since then, this part of the brain has been known as Broca’s area. It is typically defined as encompassing Brodmann areas (BA) 44 (or pars opercularis) and 45 (or pars triangularis) in the posterior half of the left inferior frontal gyrus (Ardila *et al.*, 2016; Papitto *et al.*, 2020). Importantly, Paul Broca was not able to define the exact subcortical extent of the lesions in his patients because, being aware of their historical relevance, he decided not to dissect the specimens but preserve them for future research inquiry. Broca’s descriptions therefore focused on the parts of the lesions that were visible to him (primarily at the level of the cortex) without evaluating the potential contribution of neighbouring damage, for example, to the underlying white matter and surrounding cortical areas. It was not until 2007 that the full extent of the lesions incurred by Broca’s two famous cases (Broca, 1861*a*, *b*) was revealed in an MRI study showing damage to multiple subcortical grey and white matter regions (Dronkers *et al.*, 2007).

The brain areas required for speech production, and the type of aphasia that results from damage to the posterior half of the left inferior frontal gyrus, have been continually debated since Broca’s seminal work (Marie, 1906; Mohr *et al.*, 1978; Alexander *et al.*, 1990; Lorch, 2008; Fridriksson *et al.*, 2015; Tremblay and Dick, 2016). For example, Mohr *et al.* (1978) reported that effortful speech articulation was the consequence of infarction affecting Broca’s area and neighbouring regions, including those deep in the brain. Together, these prior findings suggest that the combination of damage to Broca’s area and surrounding regions may explain persistent speech production impairments in patients with left frontal lobe strokes. Our alternative hypothesis is that persistent speech production impairments might be the consequence of damage to neighbouring regions, irrespective of the lesion status in Broca’s area. We tested these competing hypotheses, by investigating whether speech production impairments were worse in stroke survivors who had damage to: Broca’s area (i.e. BA44 and BA45) that spared surrounding regions, surrounding regions that spared Broca’s area, or both Broca’s area and surrounding regions.

Given the stereotyped distribution of vascular lesions, an ischaemic stroke will typically damage multiple neighbouring brain regions including anatomically proximal grey and white matter. Lobar haemorrhages will have a similar effect even though they do not respect vascular territories. Subcortical haemorrhages primarily affect white matter, with secondary effects (retrograde and trans-synaptic degeneration) sometimes causing later grey matter loss. In all cases, it is therefore difficult to determine which part of the lesion site is driving the observed behavioural effects (Kimberg *et al.*, 2007; Richardson *et al.*, 2012; Inoue *et al.*, 2014; Mah *et al.*, 2014; Sperber and Karnath, 2017). Here we tackled this problem in two ways. First, we studied a large number of stroke survivors who (i) all had left frontal lobe damage; (ii) differed in the degree of damage to Broca’s area and surrounding areas; and (iii) preserved posterior speech production regions in lateral inferior parietal and superior temporal association cortices. Second, having established the relative contribution of Broca’s area and neighbouring regions using multiple regression on continuous measures of structural damage (i.e. percentage of damage to each area) and speech production abilities (i.e. speech production scores), we conducted a series of *post hoc* group comparisons on small subsets of patients with distinct lesion sites.

Our selection of brain areas surrounding Broca’s area was based on a combination of anatomical and functional evidence and the availability of atlas-based regions of interest. Specifically, there are a number of long association white matter tracts that are known to link the left inferior frontal gyrus with other nodes of the speech network (Thiebaut de Schotten *et al.*, 2011; Rojkova *et al.*, 2016). Here, we exclusively focus on the following three fibre pathways: (i) the anterior segment of the arcuate fasciculus (also referred to as the third branch of the superior longitudinal fasciculus or SLF III) that connects the posterior inferior frontal cortex with the parieto-temporal cortex (Catani *et al.*, 2005; Martino *et al.*, 2013; Bernard *et al.*, 2019); (ii) the uncinate fasciculus that connects the medial and lateral orbitofrontal cortex with anterior parts of the temporal lobe (Catani and Thiebaut de Schotten, 2008; Martino *et al.*, 2011); and (iii) the frontal aslant tract that connects the posterior inferior frontal cortex with the supplementary/pre-supplementary motor area (Catani *et al.*, 2012*a*; Vergani *et al.*, 2014). In addition, we selected six grey matter regions: BA44 and BA45 (different parts of Broca’s area), the ventral premotor cortex (vPMC), primary motor cortex (M1), superior central insula and putamen. Damage to each of these white/grey matter regions has been associated with speech production impairments in prior lesion studies (Baldo *et al.*, 2011; Fridriksson *et al.*, 2013; Basilakos *et al.*, 2014; Seghier *et al.*, 2014; van Geemen *et al.*, 2014; Mirman *et al.*, 2015; Itabashi *et al.*, 2016).

Unlike previous studies, our analyses were aimed at disentangling how speech production abilities, months after a stroke centred on the left frontal lobe, were affected by damage to Broca’s area and the degree to which such effects were influenced by co-occurring damage to a specific set of neighbouring regions. Given the methodological constraints described above, it was not feasible to investigate, within the same study, all the grey or white matter regions that have previously been associated with speech production. For example, we did not examine temporal and parietal regions (Stark *et al.*, 2019; Forkel *et al.*, 2020), the internal capsule (Naeser *et al.* 1982), the medial subcallosal fasciculus or the periventricular white matter area (Naeser *et al.*, 1989). Nor did we investigate the inferior fronto-occipital fasciculus or long segment of the arcuate fasciculus because a previous well-powered lesion study was unable to establish a significant relationship between persistent speech production impairments and damage to either of these white matter tracts after controlling for damage to the anterior segment of the arcuate fasciculus (Fridriksson *et al.*, 2013). Nevertheless, we note that in our patient sample, lesion load in the anterior segment of the arcuate fasciculus was highly correlated [*r*(134) = 0.915] with lesion load in the long segment of the arcuate fasciculus, both of which were derived from the Natbrainlab atlas (Catani and Thiebaut de Schotten, 2008; Thiebaut de Schotten *et al.*, 2011). High collinearity between damage to the anterior and long segments of the arcuate fasciculus (i) is a consequence of both these tracts running in extremely close proximity in the fronto-parietal white matter above the insula (Catani *et al.*, 2005; Martino *et al.*, 2013); and (ii) makes it impossible to dissociate their effects in the current study. In light of this tight relationship, stroke damage to white matter, above the insula, in the vicinity of the anterior part of the arcuate fasciculus (aAF) is highly likely to affect fibres from both the anterior and long segments of the arcuate fasciculus, as well as other crossing white matter tracts.

In summary, Broca’s area continues to occupy a prominent position in clinical and non-clinical neuroscience (Tremblay and Dick, 2016; Fedorenko and Blank, 2020). We do not question the role that Broca’s area has been shown to play in normal speech production (Papoutsi *et al.*, 2009; Flinker *et al.*, 2015; Long *et al*., 2016; Mugler *et al.*, 2018). Our focus is on testing whether damage to Broca’s area contributes to speech production impairments that persist for at least 3 months after a left frontal lobe stroke. Although we do not investigate the effect of Broca’s area damage on speech production in the acute phase after stroke (<3 months), our study is particularly relevant for understanding clinical outcomes given that terms such as ‘Broca’s area’ and ‘Broca’s aphasia’ still dominate the clinical aphasiology literature (Hillis, 2007; Ardila, 2010). Likewise, although we do not characterize how spared brain regions functionally reorganize to compensate for the initial impact of Broca’s area damage, our findings should provide a framework to motivate and interpret lesion-site-specific studies of recovery in the future.

## Materials and methods

### Regions of interest

Three probabilistic human brain atlases that explicitly accommodate inter-subject variability in anatomy were used to define the borders of the grey and white matter regions of interest. The six grey matter regions were derived from the Brainnetome atlas (Fan *et al.*, 2016). These were: BA44, BA45, vPMC, M1, superior central insula and putamen. Particular attention was paid when defining Broca’s area, M1 and superior central insula. Specifically, BA44 and BA45 (together known as Broca’s area) were investigated individually rather than being combined into a single area, given the well-established differentiation between these two regions in terms of cyto-architecture (Amunts *et al.*, 1999), receptor-architecture (Amunts *et al.*, 2010), structural/functional connectivity (Anwander *et al.*, 2006; Margulies and Petrides, 2013) and, more importantly, function (Gough *et al.*, 2005; Klaus and Hartwigsen, 2019). For M1, we used the two (of five) M1 subregions from the Brainnetome atlas that are implicated in the motor control of the speech articulators (i.e. face, tongue and larynx). Regarding the insula, damage to both banks of the superior central sulcus centred at MNI coordinates [−36, 1, 10] has consistently been associated with speech production impairments after stroke (Dronkers, 1996; Baldo *et al.*, 2011; Chenausky *et al.*, 2020). Therefore, the two (of six) insular subregions from the Brainnetome atlas that permitted us to capture this specific subpart of the insula were selected, which is why we refer to our insula region of interest with the *ad hoc* term ‘superior central insula’.

Two (of three) white matter tracts were derived from the Natbrainlab atlas (Catani and Thiebaut de Schotten, 2008; Thiebaut de Schotten *et al.*, 2011). These were the anterior segment of the arcuate fasciculus (to index damage to aAF) and the uncinate fasciculus. The third (frontal aslant tract) was taken from the atlas developed by Rojkova *et al.* (2016) because this tract is not currently part of the Natbrainlab atlas. The preference for the Natbrainlab atlas was motivated by the fact that prior investigations that informed the current study had also used this atlas (Fridriksson *et al.*, 2013; Basilakos *et al.*, 2014). In Supplementary Table 1 we replicate our main result (i.e. Model 2 reported below) using white matter masks derived exclusively from the Rojkova *et al.* (2016) atlas.

**Figure 1:**
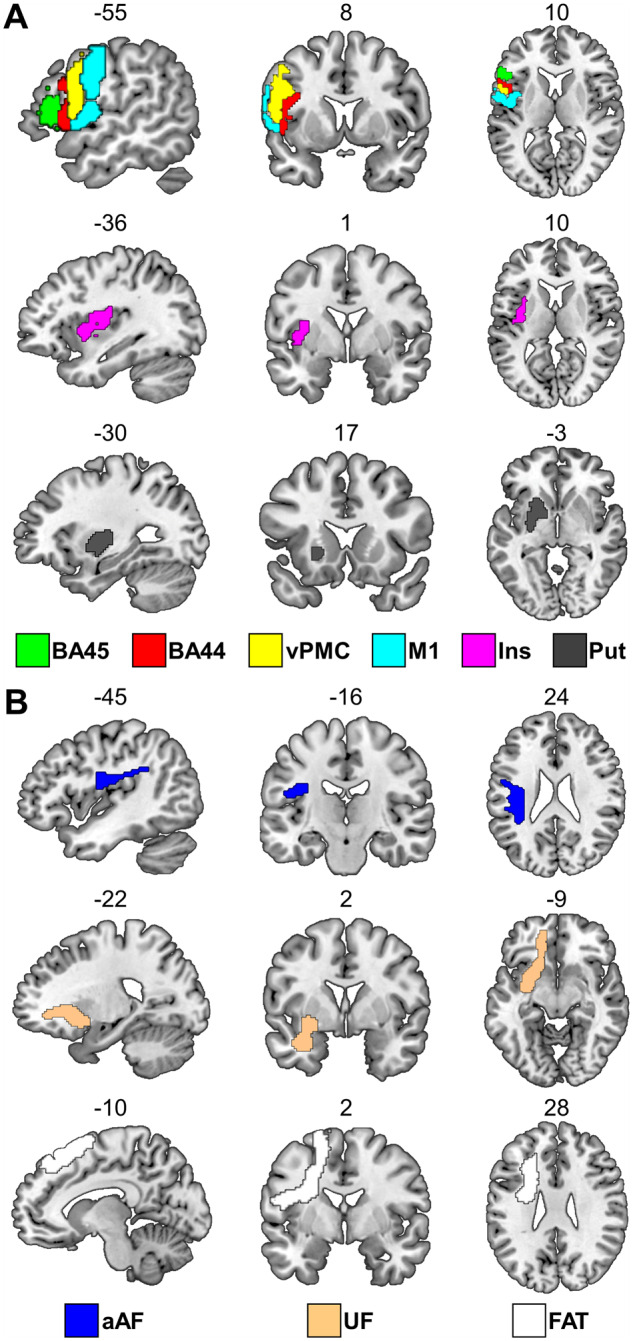
**The nine atlas-defined regions of interest.** (**A**) The *top three rows* show the six left cortical and subcortical grey matter regions of interest defined using the Brainnetome atlas (Fan *et al.*, 2016). (**B**) The *bottom three rows* show the three white matter tracts defined using tractography-based atlases of human brain connections (Catani and Thiebaut de Schotten, 2008; Thiebaut de Schotten *et al.*, 2011; Rojkova *et al.*, 2016). FAT = frontal aslant tract; Ins = superior central insula; Put = putamen; UF = uncinate fasciculus.

The borders of the regions were determined using a probability threshold of 50% for grey matter and 25% for white matter. These probability thresholds are within the range of those used in previous studies (Fridriksson *et al.*, 2013; Lunven *et al.*, 2015; Ivanova *et al.*, 2016; Hope *et al.*, 2016; Wiesen *et al.*, 2019). A probability threshold of 50% means that the anatomical localization of the region was consistent for at least 50% of the neurologically-intact participants who contributed to the construction of the atlas. A lower probability threshold for the Natbrainlab-derived white matter regions was adopted because higher probability thresholds resulted in extremely small white matter masks. See Table 1 and Fig. 1 for details.

**Table 1:** Atlas-defined regions and supporting literature for the role of the selected regions in speech production

Atlas	Left hemisphere regions of interest	Atlas label	Supporting literature
Brainnetome	BA44 BA45	IFG_L_6_1 and IFG_L_6_6 IFG_L_6_3	Broca (1861*a*, *b*); Price *et al.* (1996); Hillis *et al.* (2004); Flinker *et al.* (2015)
	Ventral premotor cortex (vPMC)	PrG_L_6_6	Wise *et al.* (1999); Price (2012); Schwartz *et al.* (2012); Seghier *et al.* (2014); van Geemen *et al.* (2014)
	Primary motor cortex (M1)	PrG_L_6_1 and PrG_L_6_5	Wildgruber *et al.* (1996); Dronkers and Ogar (2004); Price (2012); Long *et al*. (2016); Basilakos *et al.* (2015)
	Superior central insula (Ins)	INS_L_6_5 and INS_L_6_6	Dronkers (1996); Wise *et al.* (1999); Dronkers and Ogar (2004); Ackermann and Riecker (2004); Baldo *et al.* (2011); Oh *et al.* (2014); Chenausky *et al.* (2020)
	Putamen (Put)	Str_L_6_4 and Str_L_6_6	Gil Robles *et al.* (2005); Booth *et al.* (2007); Oberhuber *et al.* (2013); Seghier *et al.* (2014)
Natbrainlab	Anterior part of the arcuate fasciculus (aAF)	Anterior_Segment_Left	Catani *et al.* (2005); Marchina *et al.* (2011); Wilson *et al.* (2011); Fridriksson *et al.* (2013); Hope *et al.* (2016)
	Uncinate fasciculus (UF)	Uncinate_Left	Grossman *et al.* (2003); Papagno (2011); Catani *et al.* (2013); Basilakos *et al.* (2014)
Rojkova *et al.*	Frontal aslant tract (FAT)	Frontal_Aslant_Left	Catani *et al.* (2013); Basilakos *et al.* (2014); Mandelli *et al.* (2014); Dick *et al.* (2014, 2019)

Atlas label = labelling system used in each given atlas; Supporting literature = prior literature involving neurologically-intact controls and/or brain-damaged patients that have associated the selected brain regions with speech production.

### Patient selection criteria

Patients with an ischaemic or haemorrhagic stroke were selected from the Predicting Language Outcome and Recovery After Stroke (PLORAS) database (Seghier *et al.*, 2016), if they had unilateral damage centred on the left frontal lobe (including subcortical grey/white matter structures) as defined by a neurologist (A.P.L.). Subsequently, the T_1_-weighted whole brain image for each patient was visually inspected by A.G-V. and D.L.L-P. to rule out lesion description inaccuracies. Finally, patients whose lesions extended into posterior speech production areas in lateral inferior parietal and superior temporal association cortices were excluded from the selected sample. Inclusion criteria were: (i) aged over 18 years; (ii) no history of neurological or psychiatric illness (other than stroke); (iii) native speaker of English; (iv) right handed pre-morbidly; (v) at least 3 months post-stroke (to allow enough time for spontaneous functional reorganization to occur); and (vi) <10 years since stroke onset (to control for longer term changes related to cognitive decline).

**Figure 2:**
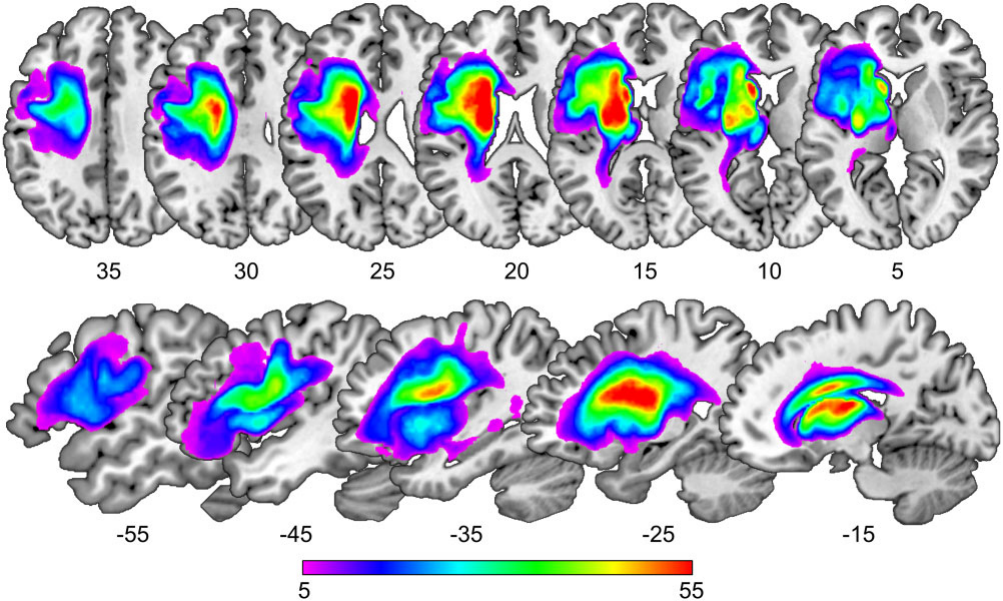
**Lesion overlap map of 134 stroke patients.** The figure shows the lesion overlap map for the full patient sample, where the colour scale depicts the frequency of overlapping lesions at each given voxel in axial and sagittal slices. Coloured areas in and around the temporal horn of the lateral ventricle indicate that our automated lesion identification procedure identified cerebrospinal fluid in enlarged ventricles as part of the lesion.

These criteria were met by 134 left-hemisphere stroke patients, aged between 31 and 87 years (mean age = 60 years). Summary demographic, clinical and lesion information for the full sample are provided in Table 2 and Fig. 2. Since 92% (123 of 134; Supplementary Fig. 1) of the patients in our sample were in the chronic phase post-stroke (>6 months), terms such as ‘long-term’, ‘long-lasting’ and ‘persistent’ throughout the current paper refer to speech production impairments that generally last longer than 6 months post-stroke. Further details are provided in Supplementary material.

**Table 2:** Summary of demographic and clinical details for all left frontal lobe stroke patients included in the study

Demographic and clinical details		Patients *n *=* *134
Age at stroke, years	Mean	57.6
	SD	12.1
	Minimum	22.8
	Maximum	85.9
Age at scan, years	Mean	60.1
	SD	12.1
	Minimum	31.4
	Maximum	87.4
Months since stroke	Mean	30.1
	SD	25.3
	Minimum	3.0
	Maximum	118.2
Total lesion volume, cm^3^	Mean	25.5
	SD	35.0
	Minimum	0.1
	Maximum	217.4
Sex	Number of females	47
	Number of males	87
SPD score	Mean	61.8
	SD	8.3
	Minimum	39
	Maximum	75

SPD = spoken picture description T-score.

The study was approved by the London Queen Square Research Ethics Committee. All patients gave written informed consent prior to participation and were compensated £10 per hour for their time.

### Behavioural assessment

All patients recruited to the PLORAS database are assessed with the Comprehensive Aphasia Test (CAT; Swinburn *et al.*, 2004). Across the 27 subtests that comprise the CAT, we selected the spoken picture description task as our behavioural index of speech production abilities, with the goal of ensuring the ecological validity of our findings by assessing connected speech production (rather than single word production) in a setting that resembled those encountered in real-world scenarios more closely. Our decision was also motivated by recent efforts to characterise the complex nature of speech production, their deficits and neural correlates using samples of connected speech (Stark *et al.*, 2019; Alyahya *et al.*, 2020; Ding *et al*., 2020). The CAT spoken picture description task was administered to each patient and the spoken responses scored following standardized procedures described in the assessment battery manual. First, the participant is shown a picture depicting a complex scene and prompted to describe verbally what is happening for 1 min without prior practice. If the patient misses out areas of the picture, the tester is required to use prompts such as ‘What about that?’. Second, the connected speech sample is scored on various properties: the total number of appropriate information carrying words (i.e. words that convey exact meaning in the appropriate context and are correctly produced) minus the total number of inappropriate information carrying words (i.e. information carrying words that are incorrectly selected and/or produced), plus syntactic variety (on a 0–6 scale), grammatical well-formedness (on a 0–6 scale) and speed of speech production (on a 0–3 scale). The summed scores are converted into a T-score. A T-score ≤60 constitutes the impaired range.

The CAT spoken picture description task is a reasonable proxy for natural speech production, because it requires patients to interpret a complex scene and report their interpretation in a coherent, free-form manner. However, there are no ‘pure’ indices of speech production. For example, in order to describe what is happening in a picture, the patient must be able to recognise the objects that are present in the scene from low-level visual features and retrieve semantic relationships between objects. Thus, to account for impairments on the CAT spoken picture description task that might be due to visual perceptual, object recognition and/or semantic processing deficits, our analyses (see below for details) factored out scores from the CAT semantic memory task. This task involves viewing a target picture (e.g. a monkey) and silently selecting one picture out of four alternatives (e.g. banana, pear, chocolate and envelope) which is most closely associated with the target.

### MRI data acquisition, preprocessing and lesion identification

T_1_-weighted high-resolution anatomical whole-brain volumes were available for all patients (*n = *134). One hundred and eleven patients underwent structural MRI at the UCL Wellcome Centre for Human Neuroimaging. The remaining 23 patients were scanned at the Birkbeck-UCL Centre for Neuroimaging. Four different research-dedicated MRI scanners (Siemens Healthcare) were used to acquire the structural images: three patients were imaged on a 3 T Prisma scanner, 78 on a 3 T Trio scanner, 30 on a 1.5 T Sonata scanner, and 23 on a 1.5 T Avanto scanner. For anatomical images acquired on the 1.5 T Avanto and 3 T Prisma scanners, a MPRAGE sequence (Mugler and Brookeman, 1990) was used to acquire 176 sagittal slices with a matrix size of 256 × 224, yielding a final spatial resolution of 1 mm isotropic voxels (repetition time/echo time/inversion time = 2730/3.57/1000 ms and 2530/3.34/1100 ms at 1.5 T and 3 T). For anatomical images acquired on the other two scanners, a MDEFT sequence (Deichmann *et al.*, 2004) was used to acquire 176 sagittal slices with a matrix size of 256 × 224, yielding a final spatial resolution of 1 mm isotropic voxels: repetition time/echo time/inversion time = 12.24/3.56/530 ms and 7.92/2.48/910 ms at 1.5 T and 3 T, respectively.

All T_1_-weighted images were converted into a binary image of the lesion in MNI space, using automated procedures reported in Seghier *et al.* (2008); see Supplementary material for more details. For each patient, the binary lesion image was visually inspected and checked against the normalized T_1_-weighted anatomical whole-brain volume/neurologist’s lesion description, and improved if necessary. The binary lesion images allowed us to delineate the lesions, to estimate total lesion volume, to generate a lesion overlap map and to compute lesion load (% damaged) in each of the nine atlas-defined regions of interest. These lesion load values were the inputs to the regression analyses described in the next section.

### Explaining long-term speech production outcome

To investigate whether inter-patient differences in speech productions abilities were significantly explained by the degree of damage to Broca’s area, surrounding brain regions or both, we applied a series of multiple regression models to the data (*n *=* *134). Using multiple regression diagnostic statistics and plots (Field, 2018), we established that our data met all core assumptions for multiple regression, with the exception of ‘high multicollinearity’ for two regressors of interest (vPMC and frontal aslant tract) and one regressor of no interest (total lesion volume). See Supplementary material for more details.

Each multiple regression model was designed to incrementally and systematically test specific aspects of our hypotheses as detailed below. The analyses were conducted in IBM SPSS Statistics for Windows, Version 22.0 (IBM Corp., Armonk, New York, USA). Comparison of correlation coefficients was achieved via Fisher’s *r*-to-*z* transformation. We used an ‘enter’ rather than ‘stepwise’ method because we were interested in testing the relative importance of individual a priori selected regions of interest rather than in identifying the best combination of regions of interest in a data-driven way. For Models 2 and 3 below, we factored out variance that was unrelated to lesion site by including the following regressors of no interest: (i) total lesion volume; (ii) months post-stroke; (iii) age at stroke; and (iv) scores from the CAT semantic memory task (to account for impairments on the CAT spoken picture description task that might be due to visual perceptual, object recognition and/or semantic processing deficits). See Supplementary Fig. 2 for a correlation matrix showing the shared variance among the different variables.

In Model 1, we tested how well speech production impairments were explained by the degree of damage to BA44 versus BA45. The two regressors of interest were lesion load in BA44 and BA45. The outcome variable was the spoken picture description (speech production) scores.

In Model 2, we excluded BA45 and added the seven remaining regions of interest into the regression (vPMC, M1, superior central insula, putamen, aAF, uncinate fasciculus and frontal aslant tract). Lesion load in BA45 was excluded because Model 1 indicated it was not a significant predictor.

In Model 3, we limited the analysis to two regressors: lesion load in BA44 and aAF, because aAF was the only significant anatomical predictor in Model 2. To test whether the effect of BA44 damage on speech production abilities was non-linear, the first version of Model 3 (i.e. Model 3a) included a quadratic term (i.e. a curvilinear relationship indicating that the greater the degree of BA44 damage the greater the detrimental effect on speech production abilities). In contrast, the second version of Model 3 (i.e. Model 3b) included an interaction term to test the possibility that the effect of BA44 damage on speech production abilities might be moderated by the degree of aAF damage. To test whether dorsal (dBA44) and ventral (vBA44) components of BA44 contribute differently to speech production, as proposed by Papoutsi *et al.* (2009), we replaced BA44 with: (i) dBA44 (i.e. Brainnetome atlas region IFG_L_6_1) in Model 3c; or (ii) vBA44 (i.e. Brainnetome atlas region IFG_L_6_6) in Model 3d.

In Model 4, lesion load in aAF was paired with either BA45 (Model 4a), BA44 (Model 4b), vPMC (Model 4c) or M1 (Model 4d) in the context of regression-based mediation analyses. This allowed us to estimate the degree to which the effect of damage to different parts of the left posterior inferior frontal cortex on our speech production scores was explained by co-occurring damage to the underlying white matter. Each of these analyses used a three-step procedure (Hayes and Rockwood, 2017, 2020) implemented in the PROCESS macro (version 3.5) for SPSS (Hayes, 2018). In Step 1, the total effect of cortical damage (e.g. to BA44) was calculated by running a regression analysis with lesion load in BA44 as the only regressor and speech production scores as the outcome variable. This produced the regression coefficient *c*. In Step 2, lesion load in BA44 was the only regressor and lesion load in aAF was the outcome variable. This produced the regression coefficient *a*. In Step 3, the regressors were lesion load in BA44 (regression coefficient *c*′ or direct effect) and lesion load in aAF (regression coefficient *b*) and the outcome variable was speech production scores. Steps 1–3 were repeated for each of the other cortical areas (BA45, vPMC and M1). The product of regression coefficients *a* (from Step 2) and *b* (from Step 3) is referred to as the indirect or mediation effect (i.e. the part of the total effect of cortical damage that is mediated by co-occurring aAF damage). The significance of the indirect effect was determined via statistical inference based on bootstrap confidence intervals (built using 10 000 bootstrap samples). See Supplementary Fig. 3 for a schematic depiction of a standard simple mediation analysis.

The goal of all our analyses was to estimate the effect of damage in regions of interest (primarily BA44 and BA45) that have previously been associated with speech production by an ample body of evidence (Table 1). In this context, we considered that the risk of making type I errors (false negatives) was greater than that of making type II errors (false positives). Put differently, based on prior evidence the unexpected result would be not to find (rather than to find) a significant relationship between damage and speech production impairments for all the regions examined. For these reasons, it would have been overly conservative to apply a correction for multiple comparisons within Model 2 (our main result), for example. Instead, we quantified the strength of the evidence in favour of the null hypothesis (no effect of Broca’s area damage) compared to the alternative hypothesis (an effect of Broca’s area damage). This requires Bayesian statistics, because frequentist approaches can only reject the null in favour of the alternative hypothesis. The Bayesian analysis reported in the ‘Results’ section was implemented in JASP (Version 0.12.2, JASP Team) using default uninformative priors (i.e. a stretched beta distribution with width = 1, which yields a uniform distribution on Pearson's *r*) because we opted to remain agnostic as to the shape of the effect size distribution.

### *Post hoc* analyses

To compare the effect of damage to (i) BA44 only; (ii) aAF only; or (iii) both, we needed to select a lesion load threshold above which a particular region would be deemed to be ‘damaged’ and control for differences in total lesion volume. Our choice was governed by the small number of patients with relatively focal damage to BA44. Within our sample of 134 patients, only eight had >20% damage to BA44 with <20% damage to aAF, 13 had >20% damage to aAF with <20% damage to BA44, and 21 had >20% damage to both aAF and BA44. Patient numbers fell when these thresholds were changed (see Supplementary Table 2 for details).

We then matched total lesion volume (range and mean) across groups. First, we excluded patients with lesions that were either smaller than the minimum (11.1 cm^3^) or larger than the maximum (62.2 cm^3^) total lesion volume in the BA44 group. Second, we matched for mean total lesion volume across groups, by excluding the patient with the smallest lesion (11.1 cm^3^) in the BA44 group and the two patients with the largest lesions (59.3 and 58.6 cm^3^) in the aAF group (see Supplementary Table 3 for details). Critically, whereas the former patient (from the BA44 group) performed within normal limits on the spoken picture description task, the latter two patients (from the aAF group) both had impaired spoken pictures description scores. Therefore, our final results would have been strengthened rather than weakened if we had not applied this strict matching procedure to ensure that total lesion volume could not explain lesion location effects. In total, there were seven, seven and eight patients who were matched for total lesion volume (range and mean) in the BA44, aAF, and BA44+aAF groups, respectively. In addition, these groups did not differ in terms of age at stroke, age at scan and time post-stroke (all *P *>* *0.45; Supplementary Table 3). All seven patients in the BA44 group and all but one (PS1129) of the seven patients in the aAF group had ischaemic strokes. Therefore, any difference in speech production scores between the BA44 and aAF groups cannot be explained by the type of stroke. See Supplementary Figs 4 and 5 for lesion location details.

The speech production scores for the BA44, aAF, and BA44+aAF groups were submitted to a one-way ANOVA. Given the small number of patients in each group, pairwise comparisons were conducted using Fisher's least significant difference (LSD) method, which does not control the family-wise error rate.

### Data availability

The data that support the findings of this study are available from C.J.P. (c.j.price@ucl.ac.uk) upon reasonable request.

## Results

### Main analyses

#### Model 1: BA44 versus BA45

Lesion load in BA44 but not BA45 significantly predicted speech production scores (Table 3). Importantly, however, this two-region model only accounted for a small proportion of the variability in speech production scores (R^2^ = 0.194, *P *<* *0.001).

**Table 3:** Results from multiple regression Models 1–3

Model	Predictors	R^2^	Adjusted R^2^	*P*-value	Beta
1		0.194	0.182	–	–
	BA45	–	–	0.395	0.101
	BA44	–	–	<0.001	−0.511
2		0.515	0.466	–	–
	BA44	–	–	0.567	0.102
	vPMC^a^	–	–	0.479^a^	−0.145^a^
	M1	–	–	0.541	0.116
	Ins	–	–	0.749	0.046
	Put	–	–	0.678	−0.053
	aAF	–	–	0.008	−0.330
	FAT^a^	–	–	0.965^a^	−0.010^a^
	UF	–	–	0.367	−0.143
3a		0.501	0.473		
	BA44	–	–	0.474	0.175
	BA44 quadratic	–	–	0.534	−0.145
	aAF	–	–	0.006	−0.295
3b		0.500	0.472		
	BA44			0.933	0.010
	aAF			0.010	−0.297
	BA44 × aAF			0.790	0.038
3c		0.500	0.476	–	–
	dBA44	–	–	0.605	0.045
	aAF	–	–	0.007	−0.290
3d		0.499	0.475	–	–
	vBA44	–	–	0.844	0.016
	aAF	–	–	0.008	−0.280

Anatomical predictors = lesion load in the atlas-defined areas for each of our 134 left frontal lobe stroke patients. Models 2 and 3 also included the following regressors of no interest: (i) total lesion volume, (ii) months post-stroke, (iii) age at stroke and (iv) scores from the semantic memory task (see Supplementary Table 4 for regressors of no interest). BA44 × aAF = interaction term; Beta = standardized beta coefficient; dBA44 = dorsal BA44; FAT = frontal aslant tract; Ins = superior central insula; Put = putamen; UF = uncinate fasciculus; vBA44 = ventral BA44.

a Regressor affected by multicollinearity; see Supplementary material for details.

As lesion load in BA45 did not make a unique contribution to the prediction of speech production scores, above and beyond that of BA44, it was excluded from Model 2 (see below).

#### Models 2 and 3: The effect of damage to other left frontal areas

When the effect of damage to regions neighbouring Broca’s area was taken into account (i.e. Model 2 in Table 3), lesion load in BA44 no longer explained speech production scores (*P = *0.567). Across all eight anatomical predictors included in Model 2, only lesion load in aAF reached statistical significance (*P = *0.008). Critically, there was not any indication of the existence of a non-linear relationship between BA44 damage and speech production abilities (i.e. the quadratic term) when aAF damage was controlled for (i.e. Model 3a in Table 3). Nor was there any evidence that the effect of BA44 damage on speech production abilities was moderated (i.e. the interaction term) by the degree of co-occurring aAF damage (i.e. Model 3b in Table 3). Moreover, these results did not change after segregating BA44 into dorsal and ventral components (i.e. Model 3c and Model 3d in Table 3). See Supplementary Table 4 for regressors of no interest.

#### Model 4: The importance of damage to aAF

Regression-based mediation analyses showed that, when considered separately (i.e. one regressor only), lesion load in each of our left posterior inferior frontal cortical regions (BA45, BA44, vPMC or M1) made a significant contribution to the prediction of speech production scores (see total effect in Table 4). However, when paired with lesion load in aAF (i.e. two regressors), each of these left posterior inferior frontal cortical regions stopped being statistically significant (see direct effect in Table 4). More importantly, these four analyses revealed that >70% of the influence of BA45, BA44, vPMC or M1 damage on speech production was mediated by co-occurring damage to aAF.

**Table 4:** Results from the mediation and correlation analyses

	BA45	BA44	vPMC	M1
**Mediation analyses (Model 4)**				
TE	−0.281	−0.436	−0.449	−0.520
DE	−0.010	−0.090	−0.073	−0.142
IE	−0.272	−0.345	−0.376	−0.378
IE/TE	**0.968**	**0.791**	**0.837**	**0.727**
**Correlation analyses**				
aAF	0.450	0.627	0.672	0.755

The upper part of the table indicates the (standardized) regression coefficients for the direct and indirect effects (DE and IE) of lesion load in each of four left posterior inferior frontal cortical regions on speech production (i.e. spoken picture description scores). The indirect effect corresponds to the part of the total effect (TE) for that particular region that is mediated by co-occurring aAF damage: e.g. (IE/TE) × 100 = 97% for BA45. The total and indirect effects for each of the four left posterior inferior frontal cortical regions examined were statistically significant. In contrast, none of the direct effects were statistically significant. The lower part of the table indicates the degree to which greater damage to aAF co-occurred with greater damage to each left posterior inferior frontal cortical region.

In addition, we found that aAF damage co-occurred significantly more with BA44 damage than BA45 damage [*r*(134) = 0.627 versus 0.450; *z *=* *3.487, *P *<* *0.001]; see bottom row of Table 4. Greater co-occurring aAF damage after BA44 damage than BA45 damage provides an explanation for the results of our first regression analysis (i.e. Model 1).

### Translating absence of evidence into evidence of absence

When the unique effect of BA44 damage on speech production scores (after covarying out lesion load in aAF, months post-stroke, age at stroke, total lesion volume and semantic memory scores) was re-expressed in terms of Bayes factors (BF), the evidence in favour of the null (i.e. damage to BA44 does not explain variance in speech production abilities) was more than eight times stronger (BF = 0.116) than that in favour of the alternative (i.e. damage to BA44 does explain variance in speech production abilities). This can be interpreted as positive evidence (Raftery, 1995) for the absence of a unique long-lasting effect of Broca’s area damage on speech production abilities. Conversely, for aAF, the evidence in favour of the alternative was more than four times stronger (BF = 4.389) than that in favour of the null. This can be interpreted as positive evidence (Raftery, 1995) for the presence of a unique long-lasting effect of aAF damage on speech production abilities.

### *Post hoc* analyses

**Figure 3:**
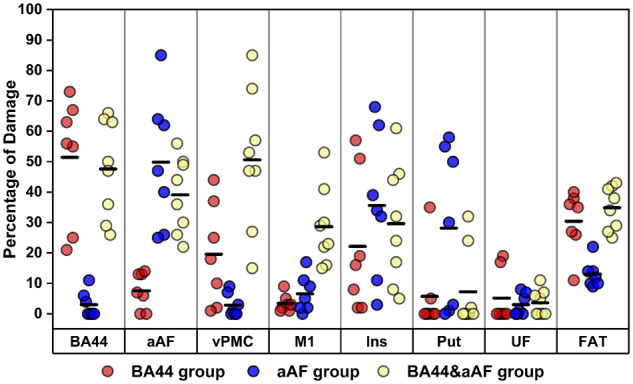
**Degree of damage to the atlas-defined regions of interest per group.** The figure shows the percentage of damage to each of the atlas-defined regions for each of the patients in each of the three groups of interest. Mean damage (per group) is represented with a thick black line. FAT = frontal aslant tract; Ins = superior central insula; Put = putamen; UF = uncinate fasciculus.

Our analyses strongly imply that damage to aAF, not BA44, is critical for explaining long-lasting impairments in speech production abilities. To illustrate this finding further, we identified three groups of patients who differed in the degree of damage to BA44 versus aAF (see ‘Materials and methods’ section). These groups had been tightly matched in terms of total lesion volume, age at stroke, age at scan and time post-stroke (all *P *>* *0.45; Supplementary Table 3). A one-way ANOVA indicated that there was a significant effect of group on speech production [*F*(2,19) = 5.028, *P = *0.018]. A Fisher’s LSD *post hoc* test showed that this occurred because the aAF group performed significantly worse than the BA44 group on the spoken picture description task [mean ± standard deviation (SD) = 55.4 ± 5.7 versus 62.9 ± 4.1; *P = *0.013], with no significant differences between the aAF group, and the BA44+aAF group (mean ± SD = 55.4 ± 5.7 versus 55.5 ± 5.3; *P = *0.979). Critically, these results did not change after covarying out inter-patient differences in lesion load in the superior central insula and putamen, which were concurrently damaged in some of the patients from the aAF group (Fig. 3 and Table 5).

**Table 5:** Results from the ANCOVA factoring out the influence of insula and putamen damage

	*F*	df	*P*-value
Main effect	4.185	2, 17	0.033

	**Adjusted means**	**Mean difference**	***P*-value**

BA44 versus aAF	62.8 versus 55.2	7.68	0.028
BA44+aAF versus aAF	55.8 versus 55.2	0.60	0.848

When considering the subscores that contribute to our aggregated speech production scores, we observed that the speech output for the aAF group was poorer both in terms of quality and quantity than that for the BA44 group as reflected in their appropriate information carrying words [mean = 19.0 versus 28.0, *t*(12) = 1.97, *P = *0.036 one-tailed], syntactic variety (mean = 3.4 versus 5.3, U = 6.5, *P = *0.009 one-tailed), grammatical well-formedness (mean = 3.6 versus 5.1, U = 11.5, *P = *0.049 one-tailed), and speed of speech production (mean = 1.6 versus 2.4, U = 8.0, *P = *0.019 one-tailed) ratings. See Supplementary Fig. 6 for the spoken picture description responses of two exemplar patients (one from the BA44 group and one from the aAF group).

A comparison of the performance of the BA44 and aAF groups across the remaining 26 tasks from the CAT is provided in Supplementary Table 5 for completeness.

## Discussion

The aim of the current study was to dissociate the effect of damage to Broca’s area and neighbouring brain regions on long-term speech production abilities in the context of relatively circumscribed left frontal lobe strokes. Our results translate into the following three novel findings: (i) Broca’s area damage does not contribute to long-term speech production outcome after relatively circumscribed left frontal lobe strokes; (ii) the long-lasting effect of damage to white matter, above the insula, in the vicinity of aAF on speech production cannot be explained in terms of a disconnection of Broca’s area; and (iii) the prior association between Broca’s area damage and long-lasting speech production impairments can be accounted for by co-occurring damage to white matter, above the insula, in the vicinity of aAF, which is highly likely to include fibres from both the anterior and long segments of the arcuate fasciculus, as well as other crossing white matter tracts.

Previous studies were not able to tease apart the impact of damage to Broca’s area and surrounding areas because focal damage to Broca’s area is rare post-stroke; particularly in the context of ischaemic injuries (Mah *et al.*, 2014; Sperber and Karnath, 2017), which are by far the most prevalent type of stroke (∼80%; James *et al.*, 2018). Indeed, none of the patients with Broca’s area damage in our sample completely preserved all neighbouring brain regions. We overcame this challenge because (i) we had access to data from a large cohort that included patients who differed in the degree of damage to neighbouring left frontal lobe regions; and (ii) although damage to Broca’s area typically co-occurs with damage to these neighbouring areas, damage to neighbouring areas can occur without damage to Broca’s area. These stereotyped vascular lesions arise because Broca’s area is fed by the precentral branch of the middle cerebral artery where a blockage proximal to its origin in the superior trunk, or at the level of the superior trunk, impacts upon neighbouring regions such as premotor and primary motor cortices (Gibo *et al.*, 1981; Kahilogullari *et al.*, 2012). In contrast, a blockage in the precentral branch distal from its origin, or in the adjacent central branch, is expected to spare Broca’s area, while affecting premotor/motor cortex and other neighbouring regions (Gibo *et al.*, 1981; Kahilogullari *et al.*, 2012).

Below, we consider prior evidence for the role of BA44 and BA45 (i.e. Broca’s area) in speech processing before turning to a discussion of why white matter damage, above the insula, in the vicinity of aAF is important for explaining long-term speech production outcome after stroke.

### The role of BA44 and BA45 in speech production in the neurologically-intact brain

BA44 and BA45 (together known as Broca’s area) still occupy a prominent position in highly influential dual-stream models of the speech network (Hickok and Poeppel, 2007; Rauschecker and Scott, 2009; Gow, 2012; Friederici and Gierhan, 2013) and the function attributed to these areas is continually being refined (Papoutsi *et al.*, 2009; Flinker *et al.*, 2015; Long *et al*., 2016; Mugler *et al.*, 2018). We are not refuting the role that Broca’s area has been shown to play in speech production in the undamaged brain, but we are challenging the long-held assumption that damage to Broca’s area contributes to long-term speech production impairments after stroke. Below, we briefly review prior findings regarding the role of BA44 and BA45 in normal speech production and the evidence we have provided that the prior association between Broca’s area damage and persistent speech production impairments can be accounted for by co-occurring damage to white matter, above the insula, in the vicinity of aAF.

According to previous transcranial magnetic stimulation and functional MRI studies of neurologically-intact subjects, BA44 and BA45 can be dissociated based on their function, with BA44 being more important for phonological processing (i.e. related to the encoding or decoding of the sound structure of words) and BA45 being more important for semantic processing (i.e. related to the meaning of words) (Poldrack *et al.*, 1999; McDermott *et al.*, 2003; Gitelman *et al.*, 2005; Gough *et al.*, 2005; Klaus and Hartwigsen, 2019). For instance, BA44—or pars opercularis—has been shown to play a key role in phonological tasks that involve monitoring, discriminating or sequencing speech sounds (Zatorre *et al.*, 1992, 1996; Demonet *et al.*, 1996; Poldrack *et al.*, 1999; Burton *et al.*, 2000). In contrast, BA45—or pars triangularis—has been associated with tasks focusing on lexical-semantic processing such as category member judgement or generation (Poldrack *et al.*, 1999; Klaus and Hartwigsen, 2019). The frontal region associated with speech articulation in functional imaging studies of the neurologically-intact brain is, by contrast, the more posterior precentral cortex (Wise *et al.*, 1999; Price, 2012).

Given the importance of BA44 and BA45 for phonological and semantic processing abilities, it would not be surprising if damage to these regions impaired speech production. On the other hand, it is also possible that speech production could be maintained or recovered if the function of BA44 and BA45 could be supported by other brain regions. In these circumstances, we might find that damage to BA44 and/or BA45 would have a transient effect on speech production abilities that weakens with time as other areas start to compensate (Hypothesis A). Alternatively, the type of processing that is important for phonologically and semantically demanding laboratory tasks may not be as important for ‘naturalistic’ speech production as required for our spoken picture description task (Hypothesis B).

Support for Hypothesis A comes from a study by Ochfeld *et al.* (2010) who reported that ischaemia in Broca’s area resulted in transient (first 48 h), rather than persistent (>6 months), speech production impairments. However, this evidence needs to be qualified by the fact that Ochfeld *et al.* did not control for the effect of co-occurring damage to surrounding areas. Therefore, it is plausible that the transient speech production impairments observed in their sample of patients were not the direct consequence of damage to Broca’s area. Support for Hypothesis B comes from a study by Tate *et al.* (2014) who showed that direct electrical stimulation of BA44 and BA45 in patients undergoing awake surgery for glioma removal disrupted phonological and semantic skills but rarely translated into a lack of speech output. As most of our patients (123 of 134 = 92%) were tested in the chronic phase after stroke (>6 months), future cross-sectional/longitudinal studies are required to establish whether Broca’s area damage contributes to early speech production impairments (i.e. within the first few months post-stroke) when the degree of damage to surrounding brain regions (particularly aAF) is accounted for.

### The importance of aAF for speech production cannot be explained by disconnection of Broca’s area

Our findings agree with prior evidence that white matter damage above the insula (Supplementary Fig. 4) can cause long-lasting speech production impairments. The white matter pathway most likely to be affected is the anterior part of the arcuate fasciculus, according to a normative DTI-based atlas of human brain connections (Catani and Thiebaut de Schotten, 2008; Thiebaut de Schotten *et al.*, 2011) and post-mortem fibre dissection studies (Martino *et al.*, 2013). However, because of how closely the anterior and long segments of the arcuate fasciculus run in the fronto-parietal white matter above the insula, we cannot distinguish whether long-lasting speech production impairments were exclusively caused by damage to one of these segments or by a combination of damage to both these segments (as well as plausibly other crossing white matter tracts in this region).

Appreciating the importance of white matter, above the insula, in the vicinity of aAF for speech production is not novel (Marchina *et al.*, 2011; Fridriksson *et al.*, 2013; Kümmerer *et al.*, 2013). The tracts in this region are sometimes claimed to be part of the so-called dorsal stream for speech processing (Parker *et al.*, 2005), and connect multiple regions implicated in speech production such as the pars opercularis, ventral precentral gyrus, supramarginal gyrus and posterior superior temporal gyrus (Catani *et al.*, 2005; Martino *et al.*, 2013; Bernard *et al.*, 2019). Thus, these dorsally located white matter tracts are thought to enable bidirectional mappings between sensory speech processing in parieto-temporal cortex and motor speech processing in posterior inferior frontal cortex, during overt and covert production of non-words, words and sentences (Saur *et al.*, 2008; Geva *et al.*, 2011; Rolheiser *et al.*, 2011; Wilson *et al.*, 2011; Kümmerer *et al.*, 2013; Fridriksson *et al.*, 2016; Ivanova *et al.*, 2016; Lorca-Puls *et al.*, 2017). Indeed, persistent speech production impairments as a consequence of stroke damage to aAF have been reported in prior studies that did (Marchina *et al.*, 2011) and did not collect (Fridriksson *et al.*, 2013) DTI data to quantify structural abnormality in white matter pathways. Speech production impairments have also been induced after aAF disruption by means of direct electrical stimulation (van Geemen *et al.*, 2014). In contrast to our work, however, these studies did not rule out the possibility that the effect of damage/disruption to aAF on speech production could be explained in terms of a disconnection of Broca’s area.

Our study extends this literature by revealing that the effect of damage to aAF on long-term speech production outcome cannot logically be explained in terms of a disconnection of Broca’s area. Specifically, Broca’s area disconnection cannot explain why patients with direct damage to Broca’s area and relative sparing of aAF had better speech production abilities than patients with damage to aAF and relative sparing of Broca’s area. In addition, our Bayesian analysis showed that Broca’s area damage did not contribute to long-term speech production outcome after factoring out co-occurring aAF damage (i.e. significantly stronger evidence in favour of the null than the alternative), which is in accord with the results from Tate *et al.* (2014) where direct electrical stimulation to Broca’s area rarely caused speech arrest. Taken together, our findings suggest that if initial speech production impairments are observed after damage to Broca’s area, with relative sparing of aAF, they are likely to resolve. In contrast, relatively circumscribed white matter damage, above the insula, in the vicinity of aAF is likely to have a long-lasting detrimental effect that may be the consequence of disrupted functional integration among the multiple regions in inferior frontal, inferior parietal and superior temporal cortices involved in the sensorimotor control of speech production (Golfinopoulos *et al.*, 2010; Schwartz *et al.*, 2012; Mirman *et al.*, 2015; Fridriksson *et al.*, 2016), irrespective of whether or not Broca’s area has been disconnected. Future longitudinal studies are needed to test these hypotheses.

The association between persistent speech production impairments and white matter damage aligns well with prior evidence suggesting that white matter damage poses a major constraint on brain plasticity (Duffau, 2014; Herbet *et al.*, 2016; Griffis *et al.*, 2019). One explanation for this is that white matter can act as a bottle neck for multiple processing tracts from multiple neural networks, all of which are affected when the bottle neck is damaged, thereby limiting resources for recovery (Griffis *et al.*, 2017).

Our findings support prior conclusions that white matter, above the insula, in the vicinity of aAF is important for speech production (Fridriksson *et al.*, 2013), but we are not claiming that this is the only brain area where damage impairs speech production. Nor are we making any claims about which cortical areas may be indirectly affected by damage to this region or the cognitive functions served by this region. It may be the case that white matter, above the insula, in the vicinity of aAF is crucial for multiple functionally distinct brain networks. For example, we found that, compared to Broca’s area damage, damage to our aAF region of interest reduced the quality and quantity of speech output in terms of its syntactic variety, grammatical well-formedness, speed and appropriate information carrying words.

### Limitations and future directions

All nine of our regions of interest were selected because they have been associated with speech production in previous lesion, direct electrical stimulation and/or functional MRI studies (Table 1). Indeed, when these regions (e.g. BA44) were considered in isolation, there was a significant association between damage and persistent speech production impairments. However, when these regions were considered in combination, the only significant predictor of long-term speech production outcome was the degree of damage to white matter, above the insula, in the vicinity of aAF. Moreover, when the effect of co-occurring damage to our aAF region of interest was controlled for, the relationship between damage and persistent speech production impairments was no longer significant for any other region. These results have a number of implications. With respect to prior (and future) lesion studies, they highlight the importance of examining focal damage to regions of interest or controlling for co-occurring damage to aAF. With respect to prior functional MRI and direct electrical stimulation studies, they suggest that the function of our regions of interest, with the exception of aAF, can be compensated for by other undamaged regions (e.g. after functional reorganization; Young *et al.*, 2020). With respect to our own findings, a number of points are worth considering further.

First, although both anatomical and functional considerations were taken into account when defining our regions of interest, it remains possible that, within each of our regions, there may be subparts that are required for speech production and subparts that are not required for speech production. In this hypothetical case, the impact of damage on speech production will depend on which subpart, but not how much, of the region has been affected. Estimating the effect of damage only in terms of lesion load in atlas-based regions of interest may therefore lead to false negative results. Future studies will need to examine the impact of damage to functionally defined subparts of our current regions of interest. For grey matter regions, the critical locus of damage could be defined as the subparts that are normally activated during speech production. For white matter regions, the critical locus of damage could be defined as the point along the length of the tract where most of the fibres have been severed (i.e. tract disconnections as opposed to tract lesion load). Previously, we proposed a method for estimating whether a tract has been severed (Hope *et al.*, 2016; see also Griffis *et al.*, 2019 for a related approach) and demonstrated that tract disconnection metrics were generally more sensitive than tract lesion load *per se*. In the current study, we circumvented the challenges associated with using lesion load in atlas-based regions of interest by including large numbers of patients with varying degrees of damage to our regions of interest. This maximizes the available variance for analysis, ensuring sufficient statistical power to detect lesion effects as reflected by the fact that a significant relationship between lesion load and speech production impairments was found when the regions were considered in isolation.

Second, white matter lesions are likely to damage fibres with a range of different cortical projections. We can therefore not be entirely sure which cortical areas are disconnected as a result of damage to our aAF region of interest. The current study, like many others (Fridriksson *et al.*, 2013; Basilakos *et al.*, 2014), attempted to constrain this problem by using regions with a high probability of being the tract of interest according to normative atlases of human brain connections (Catani *et al.*, 2012*b*; Eickhoff *et al.*, 2018). This ensured that the regions were representative of the general population, minimizing inter-subject variability without completely removing it. For studies that aim to predict outcome in new patients, greater appreciation of variability in normal and damaged white matter tracts will be required. This could be achieved with multimodal data from, for example, diffusion tensor imaging (DTI) and direct electrical stimulation studies. In addition, the availability of DTI data may be useful for determining the integrity of specific white matter tracts, particularly in the case of haemorrhagic strokes where bleeding may disrupt, but not necessarily sever, the white matter fibres running through the affected brain area. Dissociating the effect of damage to the anterior segment of the arcuate fasciculus from that to the long segment of the arcuate fasciculus is, however, an issue that DTI data would not help to resolve given the frequency with which these two segments are concurrently damaged as a consequence of stroke (at least in our dataset).

Third, there are other white matter tracts where combined stroke damage has previously been associated with persistent non-fluent speech production. These fibre pathways are more deeply situated (i.e. adjacent to the lateral ventricle) than the ones studied here and comprise the medial subcallosal fasciculus and periventricular white matter (Naeser *et al.*, 1989; Naeser and Palumbo, 1994). Currently, the definition of the course of these tracts and their cortical terminations are not yet available in any of the published tractography-based atlases of human brain connections (see Forkel *et al.*, 2014 for relevant discussion). Further research is therefore needed to (i) precisely define the nature, course and termination of these white matter tracts; (ii) assess the degree to which these tracts are damaged in patients with lesions to our regions of interest (Broca’s area and aAF); and (iii) establish whether damage to these tracts results in persistent speech production impairments when our aAF region of interest is preserved.

Finally, we note that future longitudinal structural and functional neuroimaging studies are required to investigate how neural systems for speech production change during recovery in stroke patients with relatively circumscribed damage to aAF or Broca’s area. This endeavour is likely to be extremely challenging and may not even be feasible given that stroke lesions only very rarely affect grey matter in the absence of co-occurring white matter damage and vice versa (as we have shown here).

## Conclusion

Paul Broca’s seminal work associated persistent speech production impairments with damage to the third convolution of the left frontal lobe (i.e. inferior frontal gyrus, particularly its posterior half). However, the lesion sites observed in Paul Broca’s two historic cases (Leborgne and Lelong) involved other cortical and subcortical areas neighbouring the left posterior inferior frontal gyrus, including the white matter underlying BA44 and BA45 (Dronkers *et al.*, 2007). This along with other findings (Mohr *et al.*, 1978) led to the conclusion that long-term speech production impairments are the consequence of co-occurring damage to cortical and subcortical regions in and around Broca’s area. Our results indicate that damage to BA44 and BA45 does not contribute to long-term speech production impairments after left frontal lobe strokes. As well as challenging the long established association of Broca’s area damage with persistent speech production impairments, our findings suggest that: (i) the degree of co-occurring damage to aAF should be controlled in future lesion studies of left frontal lobe function; and (ii) the association of Broca’s area damage with short-term speech production impairments (Ochfeld *et al.*, 2010) should be re-evaluated after controlling for damage to aAF.

## Supplementary Material

awaa460_Supplementary_Data

## Abbreviations

aAF =: anterior part of the arcuate fasciculus
BA =: Brodmann area
CAT =: Comprehensive Aphasia Test
M1 =: primary motor cortex
vPMC =: ventral premotor cortex
