# Supplementary material for: Damage to Broca’s area does not contribute to long-term speech production outcome after stroke
Source: Brain. 2021 Jan 31;144(3):817–32. doi: 10.1093/brain/awaa460 (PMC8041045; doi:10.1093/brain/awaa460)
Supplement: awaa460_Supplementary_Data [file awaa460_supplementary_data.pdf]

SUPPLEMENTARY MATERIAL. Damage to Broca's area does not contribute to long-term speech production outcome after stroke, by Gajardo-Vidal, Lorca-Puls *et al.*

Materials and methods

Sample composition

Nine (= 6.7%) out of the 134 patients included in the study reported a previous history of stroke. However, no clear evidence of a second, independent vascular event was found on their T1-weighed MRI scans. In Supplementary Table 1, we replicate our main result (i.e. Model 2 reported in the main text) using data from patients with no previous history of stroke exclusively (i.e. N = 125).

Regarding the times post-stroke at which patients were assessed (see Supplementary Fig. 1), 11 (= 8.2%) out of the 134 stroke survivors included in the study were in the late subacute phase post-stroke (i.e. between 3 and 6 months) and the remaining 123 (= 91.8%) were in the chronic phase post-stroke (i.e. >6 months) as defined by Bernhardt *et al*. (2017). Furthermore, within the chronic stroke survivors, 101 (= 82.1% of the total sample) were more than one year post-stroke.

Automated lesion identification

The T1-weighted images for all patients were submitted to our validated and widely adopted automated lesion identification procedure for lesion detection and delineation (Seghier *et al*., 2008). The procedure combines an optimised segmentation-normalisation routine (Ashburner and Friston, 2005; Crinion *et al*., 2007) with an outlier detection algorithm according to the fuzzy logic clustering principle (Seghier *et al*., 2007). The outlier detection algorithm is implemented in SPM8 (Wellcome Centre for Human Neuroimaging, University College London, London, UK) and assumes that a lesioned brain is an outlier in relation to normal (control) brains. The output includes two 3D lesion images in standard Montreal Neurological Institute (MNI) space, generated at a spatial resolution of 2 × 2 × 2 mm^3. The first is a fuzzy lesion image that encodes the degree of structural abnormality on a continuous scale from 0 (completely normal) to 1 (completely abnormal) at each given voxel relative to normative data drawn from a sample of 64 neurologically-intact controls. A voxel with a high degree of abnormality (i.e. a value near to 1 in the fuzzy lesion image) therefore means that its intensity in the segmented grey and white matter deviated markedly from the normal range. The second is a binary lesion image, which is simply a thresholded (i.e. lesion/no lesion) version of the fuzzy lesion image. Critically, our automated lesion identification procedure converts a scanner-sensitive raw image into a quantitative assessment of structural abnormality that is

independent of the scanner and/or sequence used (because voxel intensities are normalised with respect to those observed in neurologically-intact controls imaged on the same scanners with the same sequences).

In Supplementary Table 1, we replicate our main result (i.e. Model 2 reported in the main text) using the fuzzy (continuous) lesion images (rather than the binary lesion images) which provide an unbiased, objective quantification of the degree of structural abnormality across the whole brain relative to neurologically-intact controls. In contrast to their binary counterpart, the fuzzy lesion images do not necessitate the adoption of an arbitrary threshold to define what is damaged or not. To compute the degree of structural abnormality in every atlas-defined regions of interest, we averaged the signal indexed by the fuzzy lesion images over all voxels within each region and entered these (rather than % damaged) into the regression.

Checking the assumptions of multiple regression

For multiple regression to generate valid results that can be generalised beyond the sample at hand, the data must pass six core assumptions: (1) independence of observations; (2) linearity of relationship between the outcome variable and each of the explanatory variables; (3) homoscedasticity; (4) no high multicollinearity; (5) lack of significant outliers and influential cases; (6) normally distributed errors. To assess whether our patient data met these assumptions in the context of Model 2 (i.e. the result that guided all other analyses), we resorted to the multiple regression diagnostic statistics and plots (Field, 2018) described below.

A Durbin-Watson test statistic smaller than 1 or larger than 3 signalled violations of the independence of observations assumption. Scatterplots and partial regression plots of the relationship between the outcome variable and each of the explanatory variables were created to check for linearity. Scatterplots of standardised residuals against standardised predicted values and studentised residuals against standardised predicted values were created to check for homoscedasticity. A variance inflation factor (VIF) value for any of the regressors greater than 10 signalled the presence of high multicollinearity. Significant outliers were defined as those that were associated with residuals that departed from the mean by more than 3 standard deviations. Influential cases were defined as those that resulted in a Cook's distance value greater than 1. A P-P plot of standardised residuals was created to check for normally distributed errors.

According to these multiple regression diagnostics, our data met all core assumptions outlined above with the exception of "no high multicollinearity". Specifically, the coefficients for two regressors of interest (vPMC and FAT) and one regressor of no interest (total lesion volume) were associated with VIF values greater than 10. Multicollinearity renders the affected

regression coefficients and corresponding significance tests unreliable, but it does not compromise the validity of the regression model as a whole. In other words, it makes it difficult to assess the relative importance of the individual regressors affected by multicollinearity. Since total lesion volume was included as a control variable, we only handled multicollinearity for vPMC and FAT by re-running the regression after removing the regressors of interest with which vPMC or FAT were highly correlated (i.e. $r > 0.80$). This was achieved in two separate steps: first for vPMC (removing BA44, M1 and FAT) and then for FAT (removing BA44, vPMC and M1), which successfully lowered their VIF values well below the cut-off (to 2.4 and 5.6, respectively). Critically, both these sanity checks confirmed that aAF continued to be the only significant anatomical predictor of speech production scores (see Supplementary Table 1).

Finally, although one case (PS0571) yielded a standardised residual greater than 3, it did not exert undue influence over the model (i.e. Cook's distance = 0.11) giving us no cause for concern.

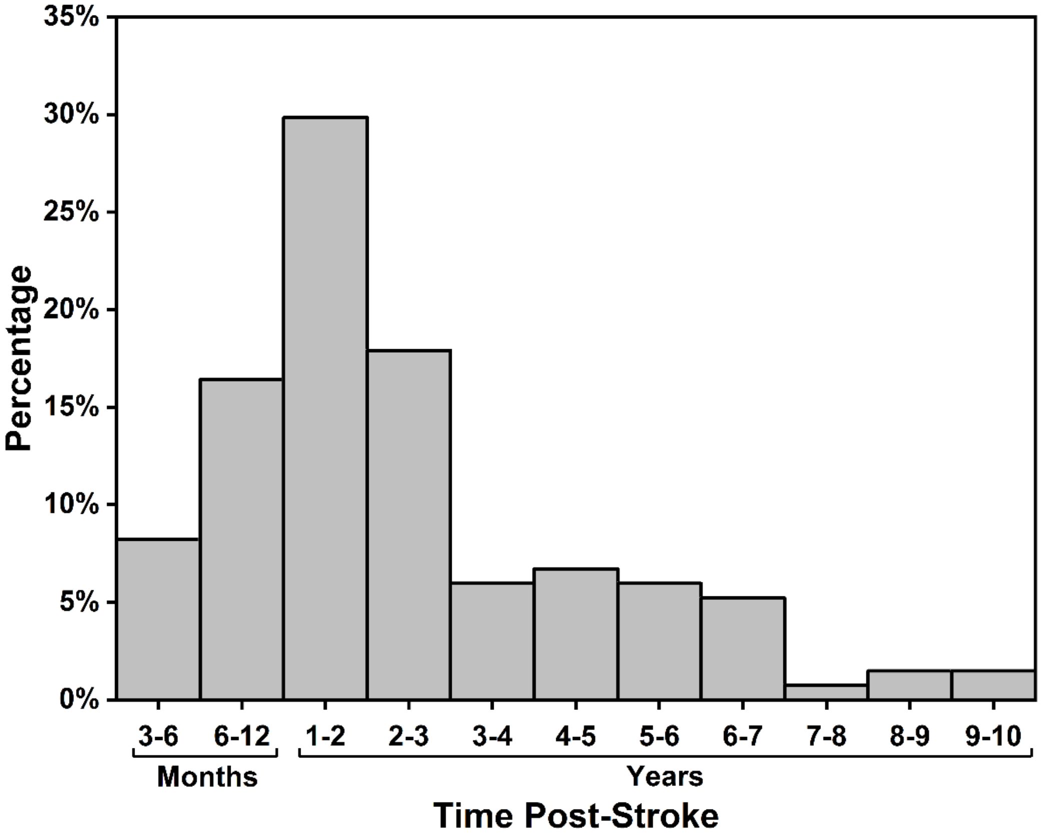

Supplementary Figure 1. Histogram of the times post-stroke at which patients were assessed.

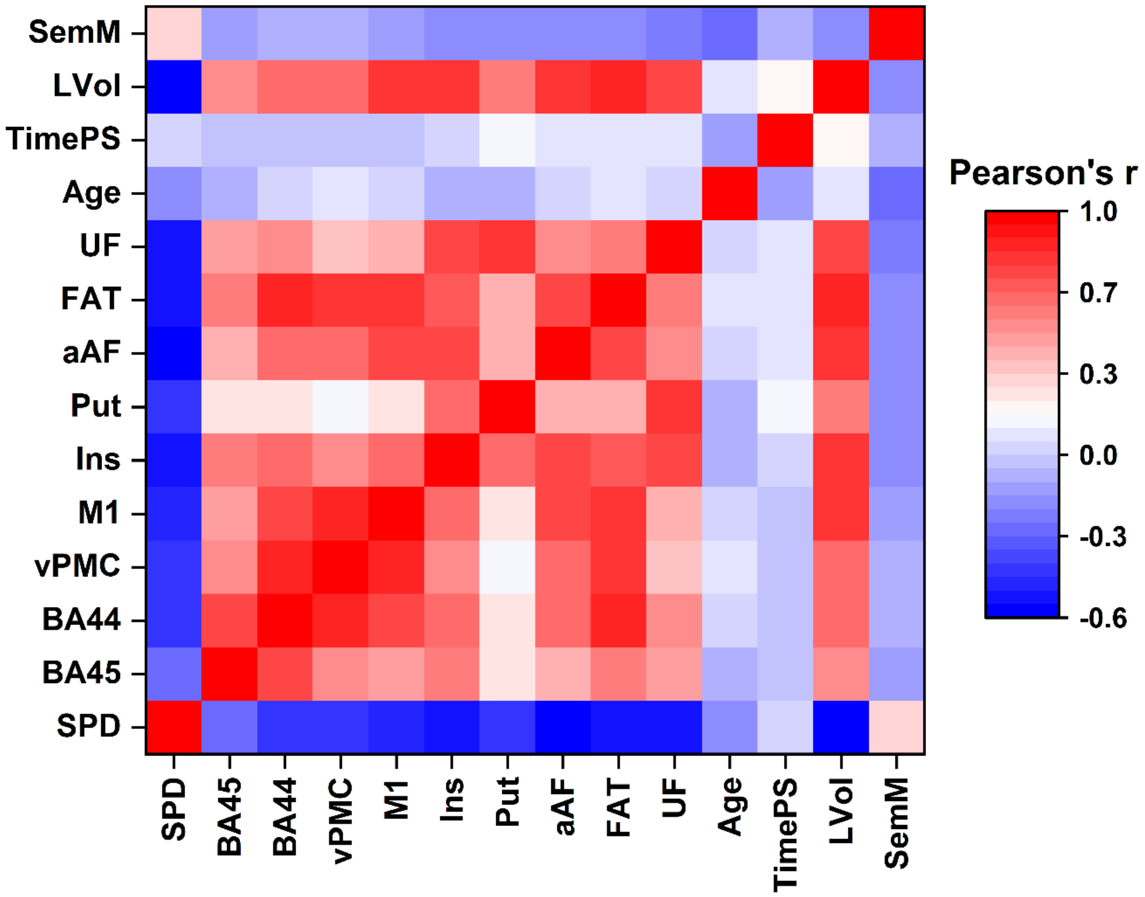

Supplementary Figure 2. Correlation matrix heatmap. Colour coded representation of the shared variance among multiple variables. **Abbreviations:** SPD = spoken picture description scores; vPMC = ventral premotor cortex; M1 = primary motor cortex; Ins = superior central insula; Put = putamen; aAF = anterior part of the arcuate fasciculus; FAT = frontal aslant tract; UF = uncinate fasciculus; Age = age at stroke; TimePS = time post-stroke; LVol = total lesion volume; SemM = semantic memory scores.

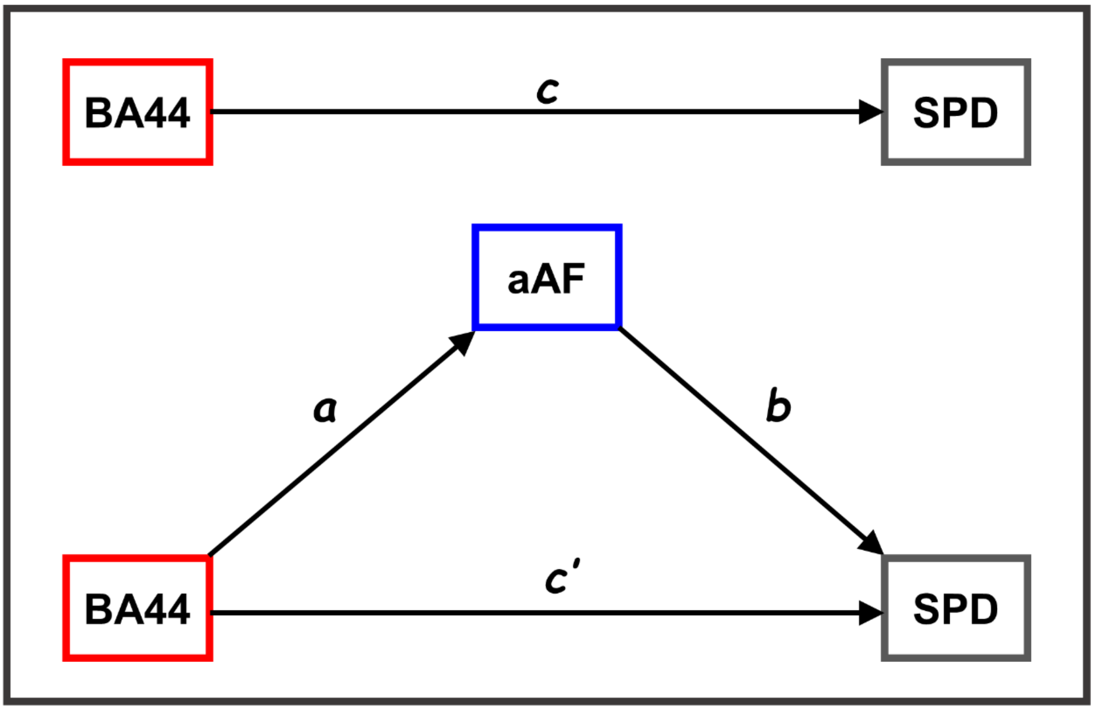

Supplementary Figure 3. Mediation model. The figure shows a schematic illustration of a simple statistical mediation analysis. BA44 = lesion load in BA44; aAF = lesion load in the anterior part of the arcuate fasciculus; SPD = spoken picture description scores; *c* = total effect (TE); *c'* = direct effect (DE); *a**b* = indirect or mediation effect (IE).

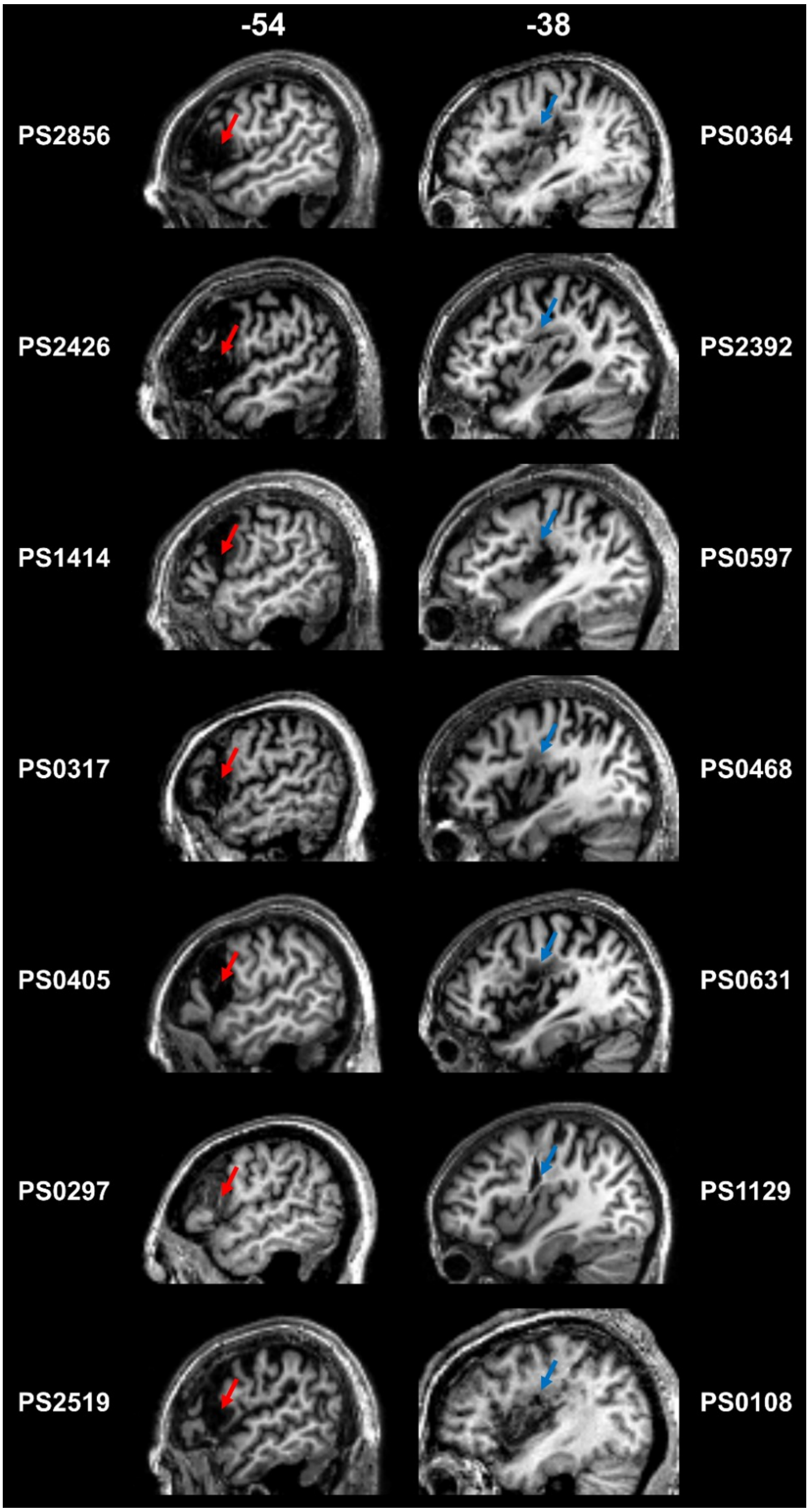

Supplementary Figure 4. T1-weighted MRI scans of patients with BA44 or aAF damage in MNI space. Sagittal views showing the stroke lesion of each individual patient from the BA44 group (left column) and aAF group (right column). Coloured arrows highlight specific brain locations at which stroke damage intersects our BA44 (red) or aAF (blue) region of interest. Next to each brain scan, patient specific identifiers are displayed.

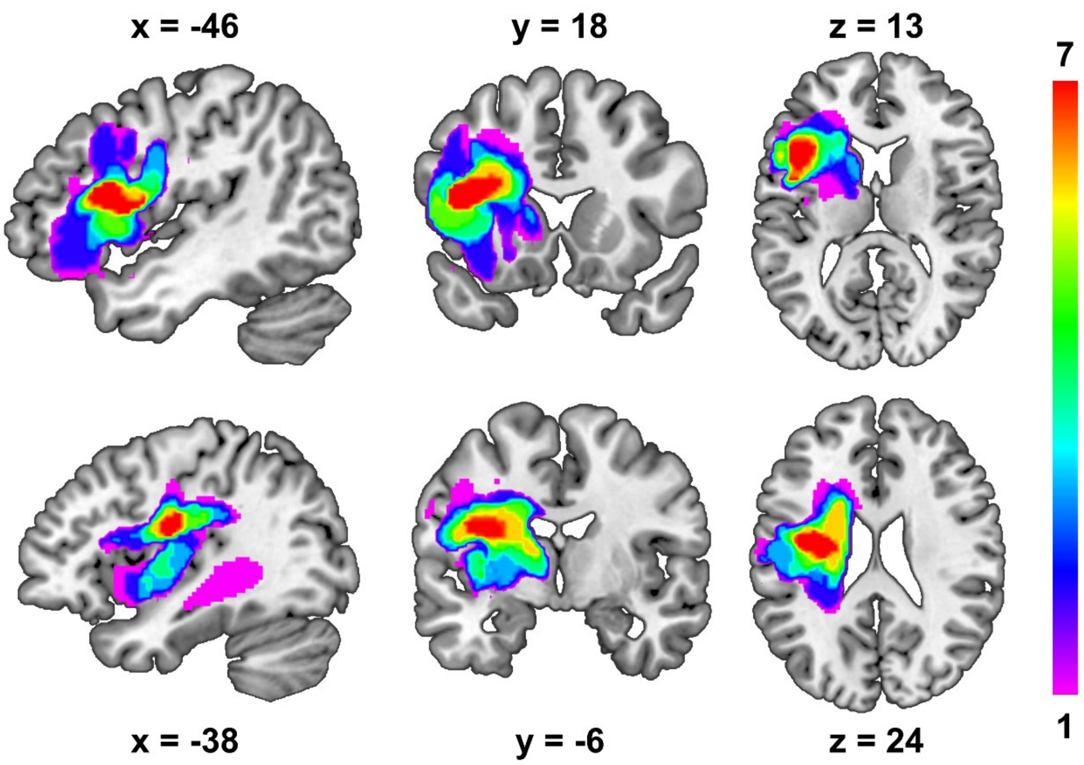

Supplementary Figure 5. Lesion overlap map of patients with BA44 or aAF damage. The top row shows the lesion overlap map of seven stroke patients with damage to BA44 and relative sparing of aAF. The bottom row shows the lesion overlap map of seven stroke patients with damage to aAF and relative sparing of BA44. The colour scale indicates the number of overlapping lesions at each given voxel. Coloured areas in and around the temporal horn of the lateral ventricle indicate that our automated lesion identification procedure identified cerebrospinal fluid in enlarged ventricles as part of the lesion.

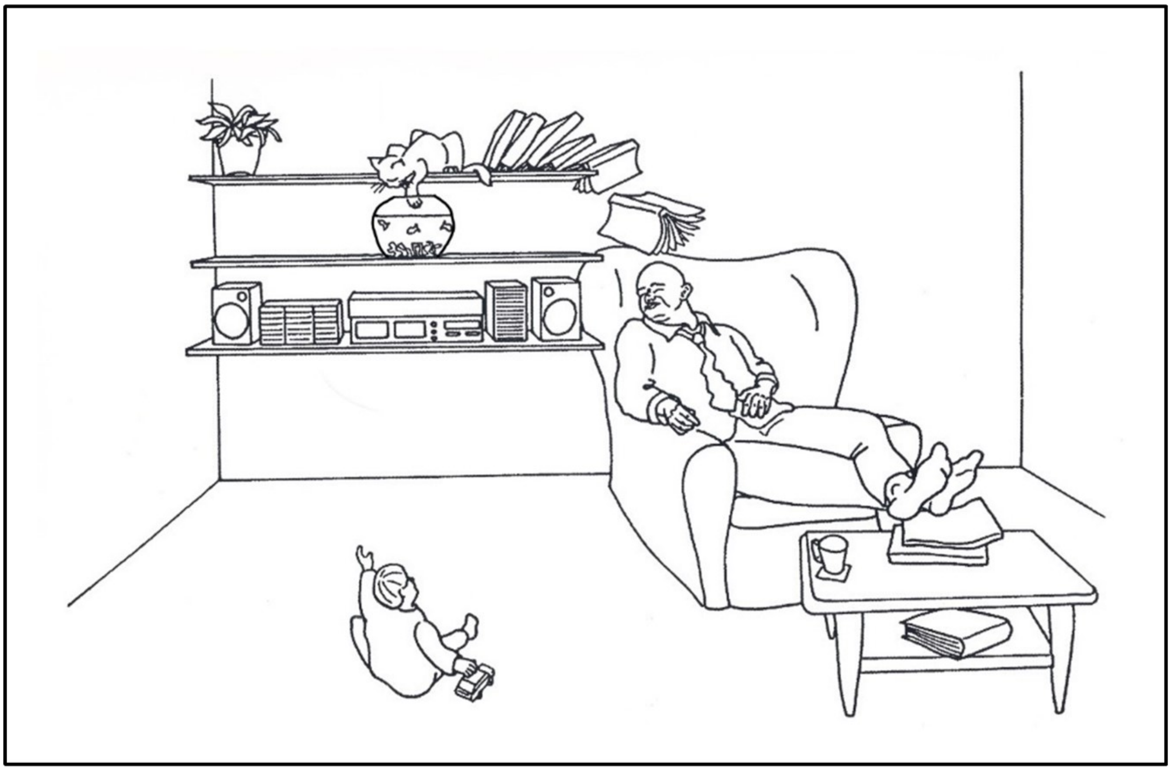

PS2856 – BA44 group

This is erm a man's sat sleeping on a chair. He hasn't got any socks on. He's got his feet on the newspaper. Erm. There's … some shelves behind him. The cat's on the top shelf, it's knocking a book which is going on the man's head. The cat's … fishing in the fish pond, in the fish tank. Erm underneath there's a stereo with some speakers. On the floor there's a little boy playing with a car. On the table where the man's feet are, there's a book underneath the table. He's got a cup 'o tea on the table. Erm, he's got a tie on this man. He's bald. **[1 minute]** Er, there's a plant on the to- on the top shelf, on the top where the cat is there's a plant. Erm, I can't think of anything else. [1 minute 12 sec]

PS0364 – aAF group

Er dentleman who a fall aseep on the … sofa … with the … di- … guy … who nee the cat on the … getting the fish and his is … er he went … [deep breath] … he went er to … tch tch tch *(pt could be gesturing something here, but this is off camera)* … erm [deep breath] di … **[1 minute]** this is who chap said … [sigh] *[Tester: "OK, so you're pointing to what's happening." Patient: "Yeah". Tester: "Yeah OK."]* [1 minute 15 sec]

Supplementary Figure 6. Transcripts of the spoken picture description responses for two exemplar patients. The picture that is shown to participants during the spoken picture description task from the CAT is provided along with the transcripts of the spoken responses for two exemplar patients from the BA44 group or aAF group. These patients had >50% damage to the BA44 (PS2856) or aAF (PS0364) regions of interest and similarly sized lesions (62.2 cm^3 and 58.2 cm^3). In PS0364 (with aAF damage), speech production is severely impaired. In contrast, PS2856 (with BA44 damage) is able to select and combine words to describe the scene in detail but misses an important point of the story (i.e. that the boy is trying to warn the sleeping man that books are about to land on his head).

Supplementary Table 1. Replication of Model 2 results

Model	Predictors	R^2	Adjusted R^2	P-value	Beta
**Using white matter tracts from the Rojkova *et al.* atlas (*N* = 134)**					
2		0.506	0.458		
	BA44†			0.929†	0.018†
	vPMC†			0.354†	-0.191†
	M1†			0.099†	0.363†
	Ins			0.791	-0.036
	Put			0.316	-0.107
	aAF			0.031	-0.353
	FAT†			0.553†	0.143†
	UF			0.537	0.088
**Using fuzzy (continuous) lesion images (*N* = 134)**					
2		0.517	0.469		
	BA44			0.892	0.025
	vPMC†			0.737†	-0.076†
	M1†			0.838†	0.043†
	Ins			0.448	0.118
	Put			0.337	-0.126
	aAF			0.006	-0.358
	FAT†			0.789†	0.067†
**Using first-ever stroke patients exclusively (*N* = 125)**					
2		0.524	0.473		
	BA44			0.514	0.118
	vPMC			0.503	-0.131
	M1			0.744	0.060
	Ins			0.737	0.050
	Put			0.733	-0.047
	aAF			0.018	-0.296
	FAT†			0.839†	-0.045†
	UF			0.375	-0.152
**Dealing with multicollinearity for vPMC (*N* = 134)**					
2		0.512	0.477		
	vPMC			0.929	-0.009
	Ins			0.566	0.077
	Put			0.550	-0.073
	aAF			0.007	-0.333
	UF			0.348	-0.135
**Dealing with multicollinearity for FAT (*N* = 134)**					
2		0.512	0.477		
	Ins			0.579	0.075
	Put			0.570	-0.070
	aAF			0.005	-0.336
	FAT			0.993	0.001
	UF			0.347	-0.135

For all replications, the following regressors of no interest were included: (i) total lesion volume, (ii) months post-stroke, (iii) age at stroke and (iv) scores from the CAT semantic memory task. † Regressor affected by multicollinearity (i.e. VIF > 10). Multicollinearity was handled by re-running the regression after removing the regressors of interest (other than aAF) with which the affected regressor was highly correlated (i.e. r > 0.80). All these sanity checks confirmed that aAF continued to be the only significant anatomical predictor of speech production scores.

Supplementary Table 2. The impact of different damage thresholds on group size

	BA44 not aAF	aAF not BA44	BA44 & aAF
Damage threshold	**>20% / <20%**	**>20% / <20%**	**>20% & >20%**
n	8	13	21
SPD score	62.6(±3.9)	54.9(±8.3)	52.7(±6.9)
Lesion volume	29.6(±19.6)	47.4(±24.6)	80.5(±48.1)
BA44	48.3(±20.9)	4.2(±4.9)	56.0(±23.0)
aAF	7.9(±5.6)	40.2(±19.8)	53.4(±19.0)
Damage threshold	**>40% / <20%**	**>40% / <20%**	**>40% & >40%**
n	5	5	13
SPD score	63.2(±4.8)	52.8(±4.8)	52.6(±7.3)
Lesion volume	39.6(±18.5)	41.3(±16.6)	87.5(±36.2)
BA44	62.8(±7.6)	6.4(±4.7)	69.3(±15.8)
aAF	9.2(±6.1)	60.6(±16.1)	57.2(±14.8)
Damage threshold	**>60% / <20%**	**>60% / <20%**	**>60% & >60%**
n	3	3	4
SPD score	60.0(±1.7)	53.7(±6.4)	47.0(±7.0)
Lesion volume	33.9(±19.1)	41.6(±14.4)	123.3(±21.4)
BA44	67.7(±5.0)	3.3(±3.1)	79.5(±12.2)
aAF	9.0(±7.8)	70.3(±12.7)	77.3(±7.3)
Damage threshold	**>80% / <20%**	**>80% / <20%**	**>80% & >80%**
n	0	1	1
SPD score	---	50.0	39.0
Lesion volume	---	58.2	105.7
BA44	---	0.0	89.0
aAF	---	85.0	84.0
Damage threshold	**100% / <20%**	**100% / <20%**	**100% & 100%**
n	0	0	0
SPD score	---	---	---
Lesion volume	---	---	---
BA44	---	---	---
aAF	---	---	---

The table shows how the number of patients (n) in each group drops rapidly with increasing damage thresholds. This empirical observation led us to choose the damage threshold that maximised between-group differences in the degree of damage to BA44 and aAF, while ensuring sufficient statistical power to match total lesion volume across groups and conduct formal statistical comparisons. Lesion volume is expressed in cm^3. Lesion load in BA44 and aAF is specified in terms of percentage of damage. Numbers indicate mean (±standard deviation). SPD = spoken picture description T-score.

Supplementary Table 3. Demographical and clinical details by group

		Lesion-volume-matched groups			Excluded from lesion-volume-matched groups		
		**BA44** n = 7	**aAF** n = 7	**BA44 & aAF** n = 8	**BA44** n = 1	**aAF** n = 6	**BA44 & aAF** n = 13
Age at stroke (years)	Mean	55.3	51.3	55.7	49.7	67.9	59.9
	SD	11.8	12.1	12.3	---	10.6	12.1
	Minimum	39.4	34.5	29.7	49.7	55.6	32.8
	Maximum	71.5	67.5	69.2	49.7	85.9	73.0
Age at scan (years)	Mean	57.9	54.5	58.0	50.7	72.8	62.4
	SD	12.3	11.7	11.4	---	8.7	12.4
	Minimum	40.7	37.2	35.1	50.7	63.3	36.5
	Maximum	74.8	68.6	70.4	50.7	87.4	79.1
Months since stroke	Mean	30.7	38.5	27.1	12.1	59.1	30.7
	SD	26.6	39.9	22.2	---	25.0	21.4
	Minimum	5.3	3.0	6.1	12.1	17.2	6.3
	Maximum	75.8	118.2	64.3	12.1	91.6	76.2
Total lesion volume (cm^3)	Mean	32.3	36.9	37.1	11.1	59.6	107.2
	SD	19.6	13.5	7.1	---	29.9	42.4
	Minimum	13.3	22.3	22.9	11.1	3.7	64.6
	Maximum	62.2	58.2	45.1	11.1	89.3	217.4
Sex	Females	4	1	4	1	1	2
	Males	3	6	4	0	5	11
SPD score	Mean	62.9	55.4	55.5	61.0	54.3	50.9
	SD	4.1	5.7	5.3	---	11.1	7.3
	Minimum	58	50	48	61	39	39
	Maximum	70	62	64	61	70	62

SPD = spoken picture description T-score. Aphasic score on SPD is 60 or below. The three selected patient groups did not significantly differ in terms of age at stroke, age at scan, months post-stroke and total lesion volume (all $p > 0.45$).

Supplementary Table 4. Regressors of no interest for Models 2 and 3

Model	Predictors	R^2	Adjusted R^2	P-value	Beta
2		0.515	0.466		
	LVol†			0.205†	-0.318†
	TimePS			0.010	0.189
	Age			0.499	-0.047
	SemM			0.018	0.166
3a		0.501	0.473		
	LVol			<0.001	-0.414
	TimePS			0.005	0.187
	Age			0.436	-0.052
	SemM			0.007	0.183
3b		0.500	0.472		
	LVol			<0.001	-0.425
	TimePS			0.006	0.184
	Age			0.428	-0.053
	SemM			0.008	0.179
3c		0.500	0.476		
	LVol			<0.001	-0.425
	TimePS			0.006	0.183
	Age			0.409	-0.055
	SemM			0.009	0.177
3d		0.499	0.475		
	LVol			<0.001	-0.413
	TimePS			0.007	0.182
	Age			0.442	-0.051
	SemM			0.007	0.181

P-values and standardised beta coefficients for the regressors of no interest: (i) LVol = total lesion volume, (ii) TimePS = time post-stroke in months, (iii) Age = age at stroke, and (iii) SemM = scores from the semantic memory task. See Table 4 for regressors of interest. † Regressor affected by multicollinearity (i.e. VIF > 10); see supplementary section entitled "Checking the assumptions of multiple regression" for details.

Supplementary Table 5. Performance across the 26 CAT tasks for BA44 and aAF groups

Task Name	Mean(±SD) by group			P-value	cut-off T-score
**Speech Production Tasks (n = 12)**	**BA44 & aAF**	**BA44**	**aAF**		
Fluency	57.8(±5.0)	60.0(±8.0)	58.9(±8.7)	0.802	57
Repetition of words	55.5(±8.3)	61.1(±6.9)	**53.7(±4.7)**	0.036	56
Repetition of complex words	55.4(±7.2)	60.6(±3.8)	56.1(±7.5)	0.199	55
Repetition of nonwords	54.0(±5.3)	62.4(±4.5)	53.6(±9.6)	0.047	51
Repetition of digit strings	56.8(±4.7)	59.9(±4.6)	57.0(±5.0)	0.286	50
Repetition of sentences	58.0(±7.1)	63.0(±0.0)	61.6(±3.8)	0.356	56
Spoken picture naming	62.5(±8.9)	66.7(±5.3)	**60.0(±8.4)**	0.099	61
Spoken action naming	56.5(±6.3)	63.6(±4.1)	**56.7(±7.3)**	0.050	59
Reading words	57.1(±6.8)	63.1(±6.1)	**56.0(±5.7)**	0.044	61
Reading complex words	58.9(±5.8)	64.7(±4.1)	59.9(±7.7)	0.167	57
Reading function words	58.8(±6.0)	62.0(±0.0)	60.6(±3.8)	0.356	48
Reading nonwords	57.6(±7.4)	65.7(±6.0)	**55.9(±12.7)**	0.098	56
**Other Tasks (n = 14)**					
Line bisection	64.3(±3.2)	59.6(±7.1)	54.0(±8.1)	0.197	39
Semantic memory	57.3(±5.2)	57.4(±4.4)	55.6(±5.7)	0.507	47
Recognition memory	56.3(±5.1)	54.6(±8.0)	55.1(±6.7)	0.887	43
Gesture object use	60.8(±5.0)	57.9(±2.7)	58.4(±5.4)	0.807	51
Arithmetic	57.4(±9.4)	58.6(±9.5)	55.3(±5.6)	0.446	40
Comprehension of spoken words	57.3(±6.5)	59.9(±5.4)	58.9(±6.4)	0.758	51
Comprehension of spoken	61.1(±5.8)	62.4(±4.5)	61.9(±5.0)	0.825	60
Comprehension of spoken	50.9(±8.0)	58.4(±4.2)	53.1(±9.0)	0.192	43
Comprehension of written words	56.9(±7.8)	57.6(±5.6)	55.6(±9.4)	0.639	53
Comprehension of written	59.0(±4.4)	64.7(±2.6)	60.1(±8.0)	0.190	57
Written copy	61.0(±0.0)	58.9(±5.7)	58.9(±5.7)	1.000	50
Written picture naming	61.5(±6.0)	63.9(±5.8)	61.0(±6.3)	0.397	54
Writing to dictation	60.4(±5.0)	63.7(±7.3)	61.1(±5.6)	0.474	57
Writing picture description	62.0(±9.4)	67.6(±4.8)	**63.4(±7.1)**	0.227	65

P-values correspond to the comparison of the BA44 group versus aAF group. Mean task scores that fell within the impaired range for the BA44 group and/or aAF group are highlighted in bold. The cut-off T-score signals the upper bound of the impaired range for that particular task. At an uncorrected statistical threshold of $p < 0.05$, the aAF group performed worse than the BA44 group on the following tasks: repetition of words, repetition of nonwords and reading words. The BA44 group did not perform worse than the aAF group on any of the tasks.

References

Ashburner J, Friston KJ. Unified segmentation. Neuroimage 2005; 26: 839-51.

Bernhardt J, Hayward KS, Kwakkel G, Ward NS, Wolf SL, Borschmann K, et al. Agreed definitions and a shared vision for new standards in stroke recovery research: the stroke recovery and rehabilitation roundtable taskforce. Neurorehabil Neural Repair 2017;31: 793-9.

Crinion J, Ashburner J, Leff A, Brett M, Price C, Friston K. Spatial normalization of lesioned brains: performance evaluation and impact on fMRI analyses. Neuroimage. 2007; 37: 866-75.

Seghier ML, Friston KJ, Price CJ. Detecting subject-specific activations using fuzzy clustering. Neuroimage 2007; 36: 594-605.
